# Predictors of breakthrough clinically significant cytomegalovirus infection during letermovir prophylaxis in high‐risk hematopoietic cell transplant recipients

**DOI:** 10.1002/iid3.431

**Published:** 2021-05-05

**Authors:** Léna Royston, Eva Royston, Stavroula Masouridi‐Levrat, Yves Chalandon, Christian Van Delden, Dionysios Neofytos

**Affiliations:** ^1^ Division of Infectious Diseases University Hospital of Geneva Geneva Switzerland; ^2^ Division of Hematology, Bone Marrow Transplant Unit University Hospital of Geneva Geneva Switzerland

**Keywords:** allogeneic hematopoietic cell transplant recipients, breakthrough infection, cytomegalovirus (CMV), letermovir, prophylaxis, risk factors

## Abstract

Letermovir prophylaxis in allogeneic hematopoietic cell transplant recipients significantly reduces the incidence of clinically significant cytomegalovirus infection. However, breakthrough infections still occur despite adequate prophylaxis. In the present retrospective cohort study, we identified clinically relevant predictive factors for clinically significant CMV breakthrough infection during letermovir prophylaxis. Low‐grade CMV replication (21–149 IU/ml), both at the time of letermovir initiation or during prophylaxis, was a significant risk factor for breakthrough clinically significant CMV infection. In addition, development of acute gastrointestinal graft‐versus‐host disease was significantly associated with breakthrough infection. Altogether these findings could call clinicians' attention to closer CMV monitoring and allow for prompt preemptive treatment initiation.

## Graphical abstract

Subclinical CMV replication and aGvHD development while on letermovir are major risk factors for CMV breakthrough infection.

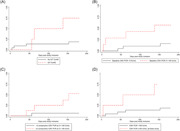

**Abbreviations**
allo‐HCT: allogeneic hematopoietic cell transplantation
CMV: cytomegalovirus
cs: clinically significant
GIT: gastrointestinal
GvHD: graft‐versus‐host disease
qPCR: quantitative polymerase‐chain reaction

## 1 INTRODUCTION

Clinically significant cytomegalovirus (csCMV) infection remains an important burden after allogeneic hematopoietic cell transplant (HCT) and contributes to significant morbidity and mortality.^1^ Due to their associated toxicities, currently used anti‐CMV agents are not routinely administered as prophylaxis and a preemptive therapeutic approach is usually followed, with treatment initiation upon CMV reactivation detection.^2^ Since January 2018, letermovir has been approved for CMV primary prophylaxis in the European Union, based on a pivotal phase‐III randomized study showing both compelling efficacy and an excellent safety profile.^3^ However, breakthrough csCMV infections during letermovir prophylaxis still occur. We performed a cohort study in our center investigating the impact of letermovir prophylaxis on allogeneic HCT recipients at high‐risk for CMV infection, in a real‐world setting.^4^ In our population, 28% of letermovir‐treated patients exhibited a breakthrough infection. In the present study, we investigated the risk factors associated with breakthrough csCMV infection.

## 2 PATIENTS AND METHODS

We included all consecutive adult allogeneic HCT recipients who received orally administered primary anti‐CMV prophylaxis with letermovir 480 mg (or 240 mg if treated with cyclosporine) once daily, between May 1st, 2019, and May 31st, 2020. The study protocol was approved by the local Ethics Committee. Based on our institutional practice, the following categories of allogeneic HCT recipients in our institution received letermovir prophylaxis during the study period: (i) CMV donor‐negative (D−)/recipient‐positive(R+) patients from post‐HCT‐day 1 to post‐HCT‐day 100 and (ii) CMV R+ patients with early (first 6 months post‐HCT) graft‐versus‐host disease (GvHD) grade ≥2 receiving corticosteroid treatment at a dose ≥1 mg/kg/day and until <10 mg/day of prednisone equivalent.^4^ All patients received antifungal prophylaxis with either fluconazole or posaconazole. Plasma‐measured CMV viremia is monitored by quantitative polymerase chain reaction (qPCR) once a week during the first 3 months post‐HCT and every other week thereafter. CMV detection is performed with COBAS® CMV for Cobas® 6800 test (Roche Diagnostics) with a limit of detection of 21 IU/ml and limit of quantification of 25 IU/ml. Based on consensus international guidelines and consistent with the pivotal letermovir clinical trial definition, we defined csCMV infection as any CMV viremia ≥150 IU/ml and/or evidence of CMV syndrome/disease requiring initiation of treatment with other‐than‐letermovir anti‐CMV agent.^3^, ^5^ Breakthrough csCMV infection was defined as an infection occurring during letermovir administration. Statistical analysis was performed using STATA 16.0 software (StataCorp). Univariable analyses were performed to identify risk factors for csCMV breakthrough infection. Identified risk factors are presented in odds ratio (OR) with 95% confidence intervals (CI). Incidence rates were calculated for variables of particular clinical relevance that were found to be statistically significant in risk factor univariable analyses.

## 3 RESULTS

**Figure 1:**
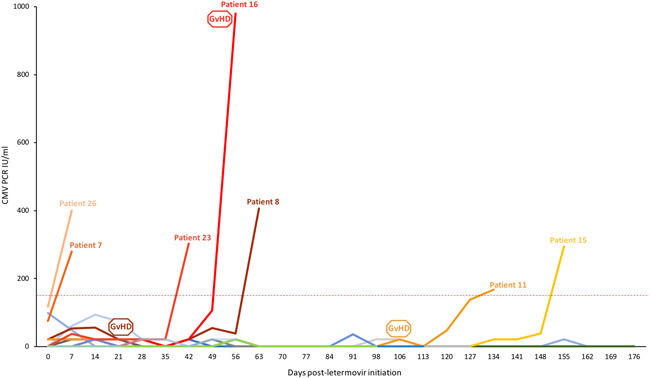
CMV viral load kinetics from letermovir initiation until end of prophylaxis, corresponding either to occurrence of breakthrough clinically significant CMV infection (>150 IU/ml), post‐HCT‐day 100 (for patients receiving letermovir for CMV donor‐negative/recipient‐positive serostatus) or concurrent corticosteroid treatment with <10 mg/day of prednisone equivalent (for CMV R+ patients receiving letermovir for early graft versus host disease (GvHD) grade ≥2). Each patient is represented by a line of different color. The horizontal red line at 150 IU/ml represents the threshold to initiate pre‐emptive anti‐CMV treatment in our institution. GvHD boxes represent the date of GvHD diagnosis for affected patients. CMV, cytomegalovirus; HCT, hematopoietic cell transplantation

**Figure 2:**
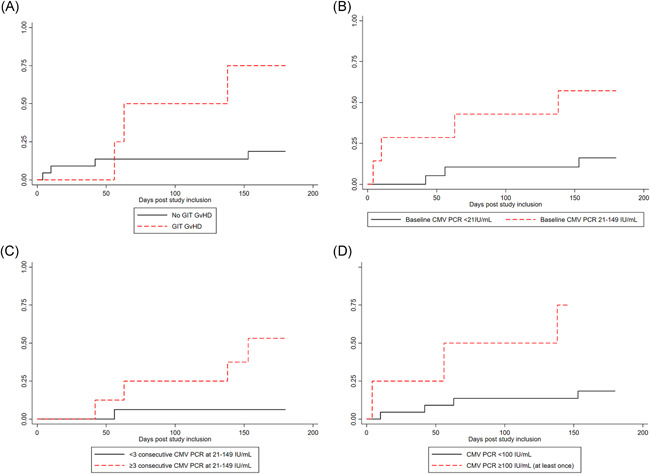
Cumulative incidence of csCMV infection in the presence (red) or absence (black) of GIT GvHD grade ≥2 diagnosis after letermovir initiation (A), of a positive CMVqPCR at the time of letermovir initiation (B), of three consecutive positive (21–149 IU/ml) CMV quantitative PCR test (CMVqPCR) (C) and of at least one CMVqPCR between 100 and 149 IU/ml (D). csCMV, clinically significant cytomegalovirus; GIT, gastrointestinal; GvHD, graft‐versus‐host‐disease

Among the 26 high‐risk HCT recipients receiving letermovir primary prophylaxis, a total of 7 (27%) patients developed breakthrough csCMV infection. Cytomegalovirus viral loads were followed from letermovir initiation until end of prophylaxis or breakthrough csCMV infection (Figure 1). Mean duration of PCR positivity after preemptive treatment initiation was of 23.8 days (range 12, 49). Patient and HCT characteristics and CMVqPCR variables were evaluated as potential predictors of breakthrough csCMV infection (Table 1). Due to the small number of cases, multivariable analyses were not performed. Patients with acute GvHD grade ≥2 affecting the gastrointestinal tract (GIT) after letermovir initiation were 13 times more likely to develop breakthrough csCMV infection (OR: 13.5; 95% CI: 1.1–166; *p* value: .04). The incidence rate of breakthrough csCMV infection during the prophylaxis period was significantly higher in patients who developed acute grade ≥2 GIT GvHD (6.9 vs. 1.2 per 1000‐patient‐days, incidence rate ratio 5.8, *p* value: .04; Figure 2A). The following CMV variables were identified as significant risk factors for breakthrough csCMV infection in univariable analyses: (i) a positive (21–149 IU/ml) baseline CMVqPCR at letermovir initiation (OR: 7.1; 95% CI: 1.0–49.5; *p* value: .05), (ii) 3 weekly consecutive CMVqPCR tests between 21 and 149 IU/ml (OR: 15.0; 95% CI: 1.3–174.4, *p* value: .03), (iii) more than three CMVqPCR tests between 21 and 149 IU/ml at any time during the study period (OR: 34.0; 95% CI: 2.4–474; *p* value: .009), and (iv) at least one positive CMVqPCR 100–149 IU/ml (OR: 13.5: 95% CI: 1.1–169; *p* value: .04). For patients with a positive CMVqPCR at letermovir initiation, the incidence rate of breakthrough csCMV infection was of 5.5 per 1000‐patient‐years (vs. 0.9/1000‐patient‐years, incidence rate ratio 5.7, *p* value: .03; Figure 2B). For patients who experienced at least 3 weekly consecutive positive CMVqPCR tests, incidence rate of breakthrough csCMV infection was 3.7 per 1000‐patient‐days (vs. 0.4 per 1000‐patient‐days, incidence rate ratio 10.0, *p* value: .03; Figure 2C). The incidence rate of breakthrough infection for patients with at least one positive PCR between 100 and 149 IU/ml was of 8.9 per 1000‐patient‐years (vs. 1.1 per 1000‐patient‐years, incidence rate ratio 7.55, *p* value: .02; Figure 2D). Among the seven breakthrough csCMV infections, no letermovir resistance‐ conferring mutation on UL56 could be identified by sequencing.

**Table 1:** Univariate analysis for clinically significant CMV infection risk factors during letermovir prophylaxis

	Odds ratio	95% CI	*p* value
*Patients characteristics and HCT variables*			
Cause of letermovir prophylaxis, D‐R+ versus GvHD	1.3	0.2, 7.5	0.78
Age	1.0	0.9, 1.1	0.63
Gender, female versus male	1.5	0.2, 9.6	0.69
Underlying hematologic malignancy, myeloid versus lymphoid	0.9	0.1, 5.8	0.88
Malignancy remission before HCT, Yes versus No	2.1	0.3, 17	0.47
Conditioning regimen, nonmyeloablative versus myeloablative	NA	NA	NA
HLA‐matched related versus HLA‐matched unrelated versus haploidentical	3.2	0.7, 14.9	0.14
Stem cells origin, bone marrow versus Peripheral blood stem cells	1.4	0.1, 18.6	0.79
Time to engraftment	1.0	0.9, 1.2	0.78
Acute GvHD grade ≥2 at baseline, Yes versus No	1.3	0.2, 7.5	0.78
Acute GvHD grade ≥2 post baseline, Yes versus No	6.4	0.8, 51.8	0.08
Refractory GvHD at baseline, Yes versus No	NA	NA	NA
GIT GvHD at baseline, Yes versus No	2.1	0.3, 12.9	0.42
GIT GvHD post baseline, Yes versus No	13.5	1.1, 166	**0.04**
CMV serological status, D+R+ versus D−R+	1.4	0.2, 9.6	0.69
EBV serological status	1.4	0.3, 6.1	0.62
Toxoplasmosis serological status	1.2	0.6, 2.5	0.57
*CMV PCR variables until end of prophylaxis*			
Positive PCR at baseline (21–149 IU/ml)	7.1	1.0, 49.5	**0.05**
Total number of positive PCR (21–149 IU/ml)	1.8	1.1, 3.1	**0.03**
2 consecutive positive PCR (21–149 IU/ml) in the first 2 weeks	3.6	0.4, 31.2	0.25
3 consecutive positive PCR (21–149 IU/ml)	15.0	1.3, 174.4	**0.03**
More than 3 positive PCR (21–149 IU/ml)	34.0	2.4, 474	**0.009**
More than 3 consecutive positive PCR (21–149 IU/ml)	12.7	1.3, 128.8	**0.03**
At least 1 positive PCR between 100 and 149 IU/ml	13.5	1.1, 169	**0.04**

Abbreviations: +, positive; −, negative; D, donor; GvHD, GIT, gastro‐intestinal tract; GvHD, graft versus host disease; HCT, hematopoietic cell transplant; R, recipient.

## 4 DISCUSSION

The first clinical trials on letermovir prophylaxis raised the hope of disposing of CMV induced complications in high‐risk allogeneic HCT recipients. However, as infections still occur despite adequate prophylaxis, it is essential to identify risk factors for breakthrough csCMV infections. To our knowledge, this is the first report attempting to identify predictive factors for breakthrough csCMV infections in allogeneic HCT recipients receiving letermovir as primary prophylaxis. We observed that early post‐HCT acute GIT GvHD significantly increased the risk of breakthrough infection. This could be due to the intensive immunosuppression associated with treatment of early GvHD, a well‐known factor for viral reactivation.^6^ Furthermore, as solely the PO formulation of letermovir was available at the time of the study, inadequate drug absorption with suboptimal drug concentrations due to GIT GvHD could also be presumed. Although currently not available, letermovir plasma drug monitoring could be particularly useful in this setting. Notably, all patients with acute GvHD before breakthrough infection had persistent low‐grade CMV replication during the weeks before GvHD diagnosis, which points to the puzzling bidirectional interaction between GvHD and CMV replication.^7^

Low‐grade viral replication was also strongly correlated with breakthrough csCMV infection. In a subgroup analysis of the pivotal letermovir clinical trial, 65% of patients with baseline viral replication at the time of letermovir initiation developed a breakthrough csCMV infection.^8^ This is consistent with our observation that baseline viral replication (between 21 and 149 IU/ml) was an independent predictor of csCMV infection amongst those allogeneic HCT recipients receiving prophylaxis with letermovir. Moreover, patients with 3 consecutive weekly measurements between 21 and 149 IU/ml were 15 times more likely to require treatment for a breakthrough csCMV infection. The range of viral replication used in this study (21–149 IU/ml) was fairly wide. This is, in part, due to our hospital virology department offering very low thresholds of detection/quantification in plasma CMVqPCR. In addition, based on our institutional standard of care, the threshold to initiate preemptive anti‐CMV treatment is 150IU/ml, likely lower than in other centers, particularly when prophylaxis with letermovir is applied, but still in the range of the threshold used for the definition of csCMV infection in the pivotal letermovir clinical trial.^3^ Due to the very small number of included patients, we were not able to study the effect of very low‐grade viremia (e.g., detectable/nonquantifiable: between 21 and 25 IU/ml or within log10: between 25 and 99 IU/ml) on the risk of breakthrough csCMV infection.

Further than the range of quantified viremia, clinical relevance of any CMV viremia under letermovir is debated, due to its unique mechanism of action. By targeting CMV‐terminase complex, letermovir prevents genome maturation, viral particle packaging and infectious virions production, but DNA synthesis is not inhibited.^9^ Based on virus isolation and DNA quantification after free‐floating DNA digestion, a recent study suggests that low‐grade (up to 1000 and 10,000 IU/ml in plasma and whole‐blood, respectively) CMV viremia detected in letermovir‐treated allogeneic HCT recipients could be mainly attributed to abortive infections and would not represent real csCMV infection events.^10^ As such, CMV DNA detection and quantification might thus underestimate letermovir efficacy and lead to excessive CMV‐preemptive treatment.^10^ While these findings are intriguing and worthy of more research, both the exponential CMVqPCR rise in most patients with breakthrough csCMV infection and the occasional occurrence of histologically proven CMV end‐organ disease in letermovir‐treated patients in the pivotal study raise concerns about real breakthrough infections.^3^ Monitoring of not yet validated other viral surrogates‐as pp67 messenger RNA‐could help to address this question, but this was not the focus of our study.^11^ Larger studies will be required to shed some more light on the significance and impact of low‐grade viral replication in patients under letermovir prophylaxis and the definition of a relevant threshold for preemptive‐treatment initiation in the setting.

In conclusion, this study highlights distinct predictors for breakthrough‐letermovir csCMV infection. Our findings suggest that low‐grade CMV replication constitutes a major risk factor for csCMV infection despite letermovir prophylaxis and emphasizes the importance of a close CMVqPCR monitoring. Gastrointestinal inflammation in the context of GvHD also enhances the risk of breakthrough infection, possibly by preventing drug absorption. These findings may constitute the basis for future research to assess the clinical significance of low‐grade CMV replication and threshold of preemptive CMV treatment while on letermovir.

## CONFLICT OF INTERESTS

Dionysios Neofytos has received research support from MSD and Pfizer, and consulting fees from Roche Diagnostics, MSD, Pfizer, Basilea, and Gilead. Yves Chalandon has received consulting fees from MSD. Léna Royston, Eva Royston, Stavroula Masouridi‐Levrat, and Christian Van Delden have no conflicts of interest.

## AUTHOR CONTRIBUTIONS

Léna Royston and Dionysios Neofytos conceived and designed the study. Léna Royston and Eva Royston collected the data. Léna Royston, Eva Royston, and Dionysios Neofytos analyzed the data. Stavroula Masouridi‐Levrat, Yves Chalandon, and Christian Van Delden provided resources. Léna Royston wrote the original draft and Eva Royston, Stavroula Masouridi‐Levrat, Yves Chalandon, Christian Van Delden, and Dionysios Neofytos revised the manuscript. Dionysios Neofytos administrated and supervised the study.

## Data Availability

Data available on request from the authors.
